# A controlled ac Stark echo for quantum memories

**DOI:** 10.1038/s41598-017-08051-5

**Published:** 2017-08-09

**Authors:** Byoung S. Ham

**Affiliations:** Center for Photon Information Processing, and School of Electrical Engineering and Computer Science, Gwangju Institute of Science and Technology, 123 Chumdangwagi-ro, Buk-gu, Gwangju 61005 South Korea

## Abstract

A quantum memory protocol of controlled ac Stark echoes (CASE) based on a double rephasing photon echo scheme via controlled Rabi flopping is proposed. The double rephasing scheme of photon echoes inherently satisfies the no-population inversion requirement for quantum memories, but the resultant absorptive echo remains a fundamental problem. Herein, it is reported that the first echo in the double rephasing scheme can be dynamically controlled so that it does not affect the second echo, which is accomplished by using unbalanced ac Stark shifts. Then, the second echo is coherently controlled to be emissive via controlled coherence conversion. Finally a near perfect ultralong CASE is presented using a backward echo scheme. Compared with other methods such as dc Stark echoes, the present protocol is all-optical with advantages of wavelength-selective dynamic control of quantum processing for erasing, buffering, and channel multiplexing.

## Introduction

In the 1980s and 1990s, photon echoes were intensively studied with regard to all-optical information processing. In an inhomogeneously broadened two-level optical system, an optical π-pulse induces population swapping between the ground and excited states, resulting in reversible coherence evolutions of an ensemble. Owing to the reversibility of coherence evolutions photon echoes inherently satisfy unitary evolution of quantum mechanics. However, the π-pulse-induced population swapping in photon echoes results in a population inversion for weak data pulses, Thus, direct use of photon echoes has been strictly limited to quantum memories due to spontaneous- and/or stimulated emission-caused quantum noises, where duplication of an unknown quantum state is strictly prohibited in quantum mechanics. To overcome the population inversion constraint in photon echoes, several modified photon echo schemes have been developed for quantum memory applications since 2001^1^. The modified photon echo schemes for quantum memory protocols include controlled reversible inhomogeneous broadening (CRIB)^1–5^ and atomic frequency comb (AFC) echoes^6–9^ for a single rephasing scheme, and silent echoes^10, 11^, dc Stark echoes^12, 13^, controlled double rephasing (CDR) echoes^14, 15^ and optically locked photon echoes^16^ for a double rephaisng scheme. These modified photon echo protocols can also be categorized as two-level^3, 4, 6, 9–13^ or three-level^1, 2, 5, 7, 8, 14–16^ schemes.

Although the population inversion constraint is removed automatically in the double rephaisng photon echo scheme, the intrinsic absorptive coherence of the final (second) echo^10–13^ has been a fundamental issue preventing its implementations (see the Supplementary Information Fig. S1). The absorptive echo in either a single^7, 8^ or double rephasing scheme^10–13^, however, has been solved by a quantum coherence control, known as controlled coherence conversion (CCC)^17^. Here CCC is a simple optical Rabi flopping achieved by a consecutive optical π-π pulses (or a single 2π pulse) resonant between the excited and a third (metastable or isolated) states. Unlike double rephasing (or Rabi flopping by a 2π pulse) in the two-level system, Rabi flopping by CCC in a three-level system induces a coherence inversion to the ensemble (see the Supplementary Information Fig. S2). Thus, the absorptive echo in the double rephasing scheme can be converted into an emissive one. Such a CCC-based double rephasing quantum memory protocol is called a CDR echo, where the first echo is supposed to be silent (see the Supplementary Information Figs S3 and S4 for a single C-pulse version). Recently the CDR echo protocol has been fully discussed using numerical^15^, analytical^18^, and Maxwell-Bloch^19^ approaches, where atom phase control by CCC is essential. In addition, both near perfect retrieval efficiency and storage time extension are additional benefits of CDR, when consecutive two π-C pulses are involved (discussed in Section F). These additional benefits are critical in various quantum memory applications such as fault-tolerant quantum computing^20^ and long-distance quantum communications^21^.

According to the analysis of double rephasing photon echoes (see the Supplementary Information Fig. S1), there is no way to obtain an emissive photon echo in a two-level system without population inversion. The recent echo observations in double rephasing schemes^10–13^, however, are due to coherence leakage from imperfect or nonuniform rephasing by a Gaussian light pulse in a transverse spatial mode perpendicular (x- and y-axis) to the beam propagation direction (z-axis)^22^. As a result echoes are always generated regardless of the rephasing pulse area in the Gaussian pulse-based experiments, where its maximum efficiency reaches 26% for a π/2−π/2 rephasing pulse sequence^22^.

In the double rephasing scheme, silencing the first echo is an essential requirement to avoid effects on the second echo. The first idea proposed for a silent echo is to use phase mismatching between the data and rephasing pulses^10^. In that scheme, the backward rephasing pulse may not fully rephase the data-excited coherence due to the Beer’s law-governed opposite absorption profile. The second idea is to use dc Stark effects with spectral tailoring^12, 13^. Here, we present a new quantum memory protocol of *controlled ac Stark echoes* (CASE) based on CDR, where unbalanced ac Stark fields work to silence the first echo so that it does not affect the second echo. The most important advantage of using ac Stark fields is in the all-optical dynamic control of quantum information. Compared with previous protocols^10–16^, the technical advances of CASE are its ease of use in handling cavity coupling difficulties due to flexible bandwidth, freedom from the phase matching condition between data and rephasing, no need for spectral tailoring, and its free access to multiple spectral channels for dynamic multiplexing.

Recently demonstrated ac Stark modulations in a double rephasing photon echo scheme^23^ have exhibited a similar mechanism of atom phase control to dc Stark echoes^12, 13^, in which the Stark field provides an on-purpose phase shift (turbulence) so that the transient atoms cannot be concurrently rephased for the first echo. As mentioned above, however, ref. 23. cannot be applied to a quantum memory protocol due to the absorptive coherence. Here, I present CASE combined by CCC with the ac Stark modulations in a double rephaisng photon echo scheme. Unlike other photon echo-based quantum memory protocols^3–13^, CASE offers potential benefits of both near perfect retrieval efficiency and ultralong storage time. Compared with the dc Stark echo protocol^3–5^, requiring high electric fields of the order of ~kV in a ~cm-long bulky (rare-earth-doped) medium^23^, the present CASE is not limited by the physical length, resulting in naturally all-optical processing compatible with ultrafast, multimode nano-photonics^24^.

The detailed characteristics of CASE are as follows. First, the gradient field required in dc Stark echoes is now optional due to a large detuning *Δ*_*AC*_, and spectral preparation can be used for multi-channel operations. Second, with a large optical detuning *Δ*_*AC*_, the ac Stark pulse-induced absorption that otherwise can affect echo efficiency can be minimized. Third, the detuning selectivity of the ac Stark fields allows for multiple channel accessibility in the frequency domain, as is the case for the wavelength division multiplexing of fiber-optic communications, where this feature can offer multi-channel manipulations of multi-spectral mode quantum memories. Fourth, an unknown quantum state to be stored or retrieved can be dynamically manipulated for an eraser^23^ or entangled photon-pair generations^25^. Fifth, CASE is immune to spectral tailoring required in dc Stark echoes. Here, the spectral tailoring is for on-demand control of spectral broadening^12, 13^. For the wavelength division multiplexing, the original wide-bandwidth ensemble needs to be divided into many spectral channels of narrow bandwidth (${{\rm{\Delta }}}_{inh}^{^{\prime} }$), where a lengthened dephasing time (${\rm{\delta }}{\rm{T}}^{\prime} =1/{{\rm{\Delta }}}_{inh}^{^{\prime} }$) normally deteriorates echo efficiency. However, the ac Stark effect does not (will be discussed elsewhere). Sixth, the storage time can be extended up to spin population-decay time by using either dynamic decoupling^26^ or optical locking techniques (discussed in Section F)^16^.

## Results and Discussion

**Figure 1:**
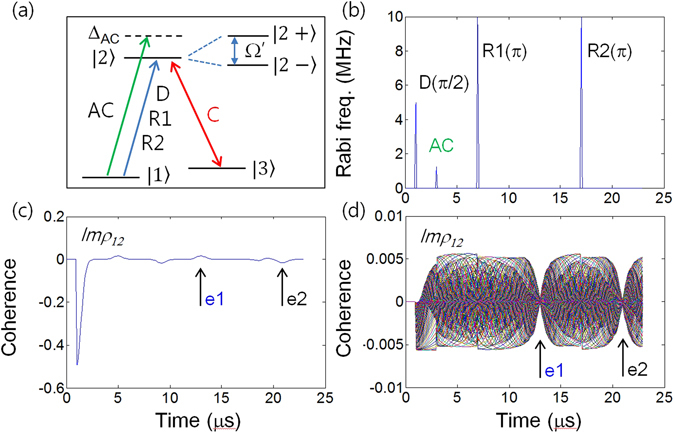
Echo erasing by ac Stark shift. (**a**) Energy level diagram for ac Stark modulation: ac Stark (AC), Data (D), Rephasing (R1), and Rephasing (R2). The control pulse C and state $|3\rangle$ are for CASE (discussed in Section D). (**b**) Pulse sequence for (**a**). (**c**) Numerical results of echo erasing (silence) for a π/2 pulse area of ac Stark field AC. (**d**) Individual atom coherence of (**c**). For this the optical inhomogeneous broadening of the two-level system is 1.7 MHz (FWHM) and is divided into 201 groups of atoms at 10 kHz spacing for individual calculations. All decay rates are set to zero. Initially, all atoms are in the ground state: ρ_11_(0) = 1. Each pulse duration is set to 0.1 μs. The time of arrival of D, AC, R1 and R2 are 1, 3, 7, and 17 μs, respectively. The AC Rabi frequency is Ω_AC_ = 1.25 MHz, and its detuning is Δ_AC_ = $\sqrt{15}{\Omega }_{AC}$. The ac Stark shift Δ_S_ is replaced by the generalized Rabi frequency of the ac Stark field ${\rm{\Omega }}^{\prime} =\sqrt{{{\rm{\Delta }}}_{AC}^{2}+{{\rm{\Omega }}}_{AC}^{2}}$. This replacement does not alter the ac Stark physics but accelerates the phase shift time. The e1 is the target echo for erasing. All frequency parameters are multiplied by 2π for all figures. AC field in (**a**) dresses both ground and excited states in the same way, where the ground state splitting is omitted for simplicity^28^.

**Figure 2:**
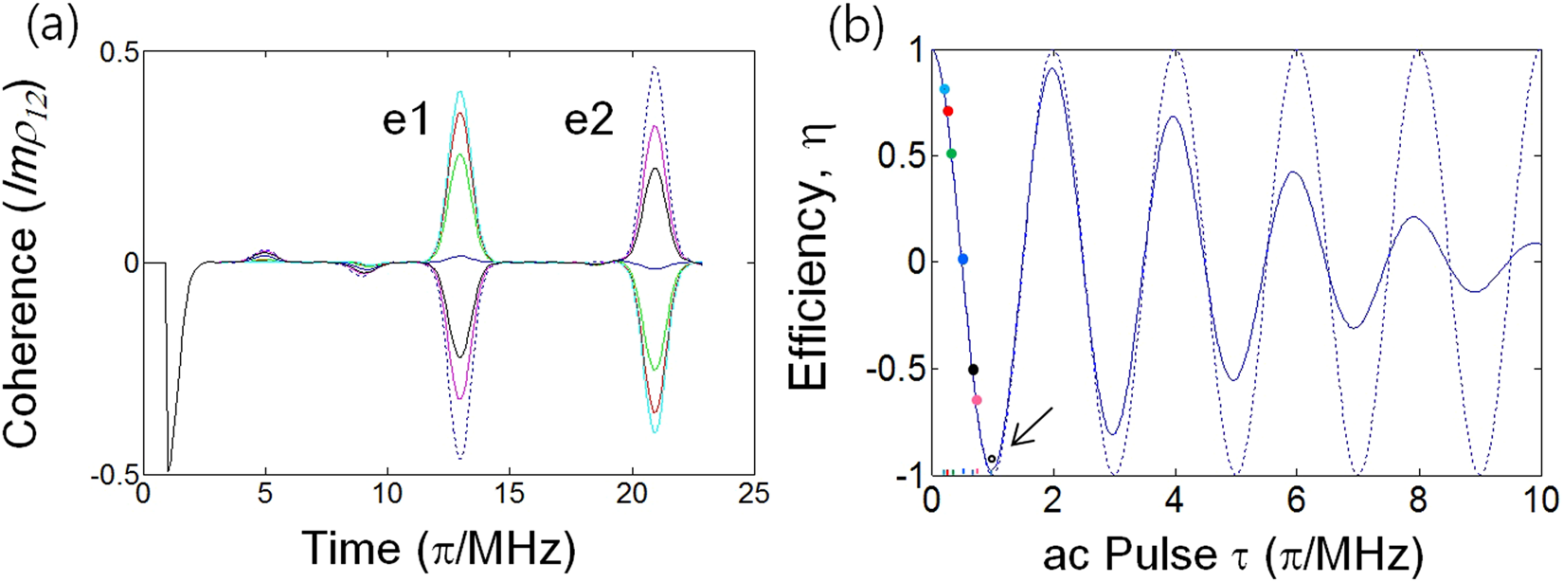
ac Stark influence on photon echo efficiency. The pulse sequence for (**a**) is in Fig. 1b. e1 and e2 are the echoes by R1 and R2, respectively. All parameters are the same as in Fig. 1, unless otherwise specified. The generalized ac Stark Rabi frequency is ${\rm{\Omega }}^{\prime} =1.02\,MHz$, where ${\Omega }_{AC}=1\,MHz$ and ${\Delta }_{AC}=5\,MHz$. (**a**) The ac Stark photon echoes for different ac Stark Rabi frequencies, $\frac{{\Omega ^{\prime} }_{AC}}{N}$, with a fixed pulse duration of *τ* = 0.1 *μs*. For ${\Phi }_{AC}=\Omega ^{\prime} \tau$, the different curves are as follows: cyan (N = 5; *Φ*_*AC*_ = *π*/5); red (N = 4; *Φ*_*AC*_ = *π*/4); green (N = 3; *Φ*_*AC*_ = *π*/3); blue (N = 2; *Φ*_*AC*_ = *π*/2); black (N = 3/2; *Φ*_*AC*_ = 2*π*/3); magenta (N = 4/3; *Φ*_*AC*_ = 3*π*/4); dashed (N = 1; *Φ*_*AC*_ = *π*). (**b**) The ac Stark echo efficiency model based on equation (1). The solid curve is the overall echo efficiency η for Eq. (1), where ${\rm{\eta }}=\sum _{j}{c}_{j}\,\cos ({{\rm{\Phi }}}_{AC}^{j})$ and the *c*_*j*_ are the weight factors for Gaussian distribution. The colored dots are the results from (**a**) for different Φ_AC_. The dotted curve is a reference for the resonant atom (δ_0_) at line center.

**Figure 3:**
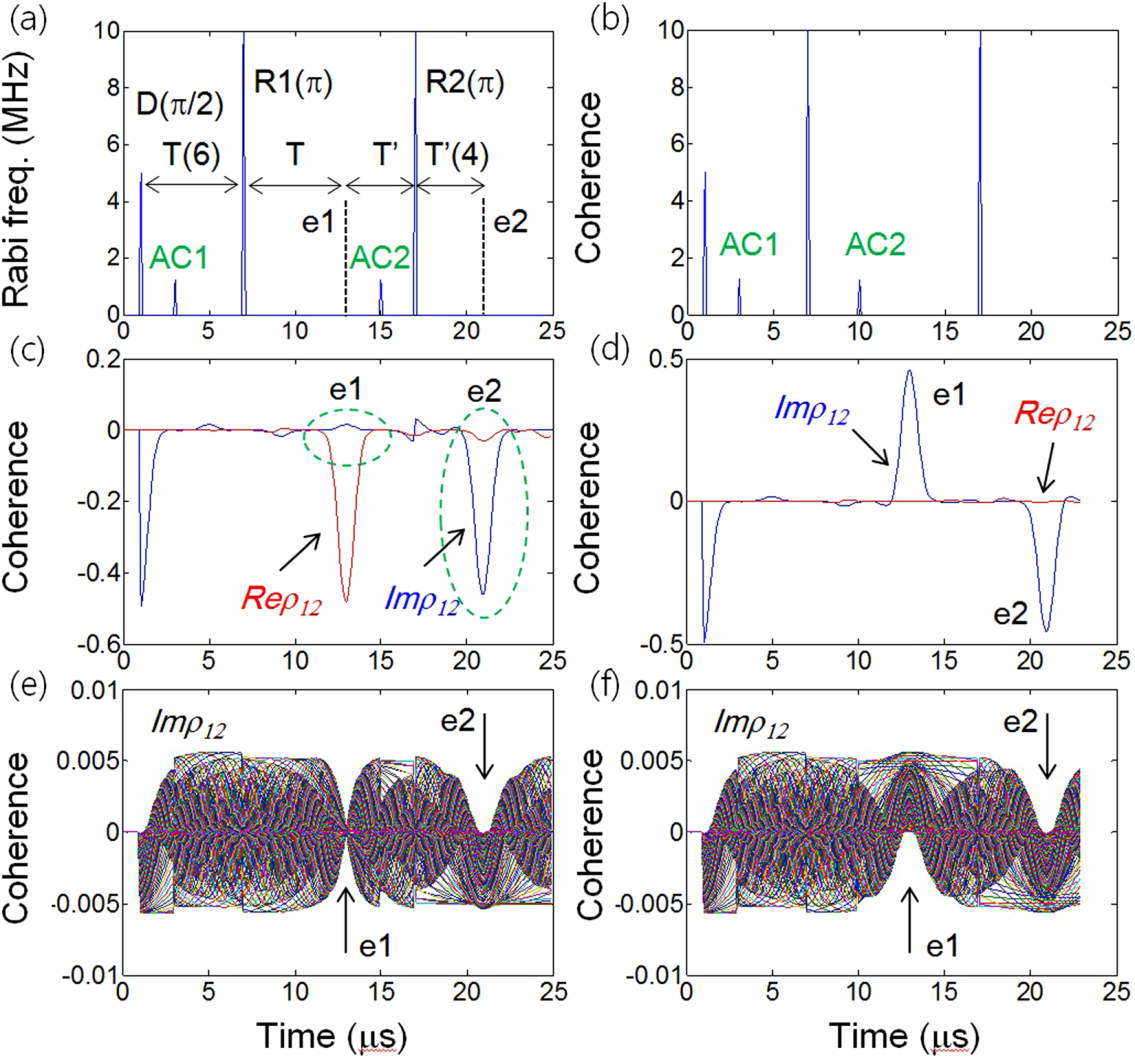
ac Stark control in doubly rephased photon echoes. (**a**),(**b**) Pulse sequence: Data (D), Rephasing (R1), Rephasing (R2), ac Stark pulses (AC1) and (AC2). The numbers T and T’ represent the time durations in μs, and the e1 (e2) timing is 13 (21) μs. (**c**–**f**) Numerical calculations for optical coherence based on the time-dependent density matrix equations. (**c**) and (**e**) are the results for (**a**), whereas (**d**) and (**f**) are for (**b**). The time of arrival of D, AC1, R1, and R2 are 1, 3, 7 and 17 μs, respectively. The arrival time of AC2 is (**a**) 15 μs and (**b**) 10 μs. The other parameters are the same as in Fig. 1, unless otherwise specified.

**Figure 4:**
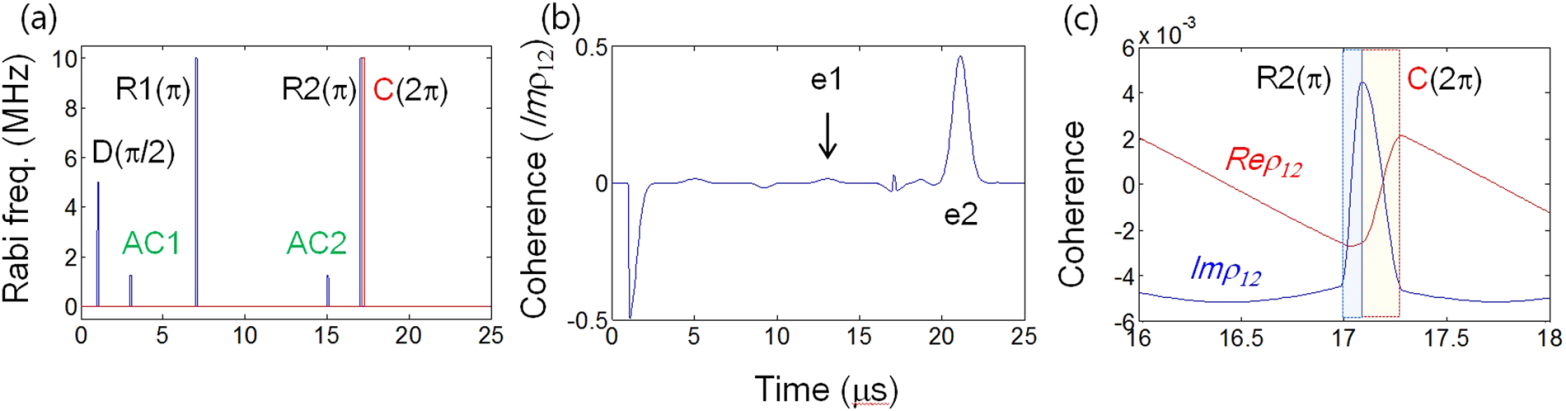
Controlled ac Stark echoes (CASE). (**a**) Pulse sequence: Data D, Rephasing R1, Rephasing R2, ac Stark pulses AC1 & AC2, and control pulse C. (**b**) and (**c**) Numerical results of (**a**). The arrival time of C is 17.1 μs with 0.2 μs pulse duration. All parameters are the same as in Fig. 3, unless otherwise specified.

**Figure 5:**
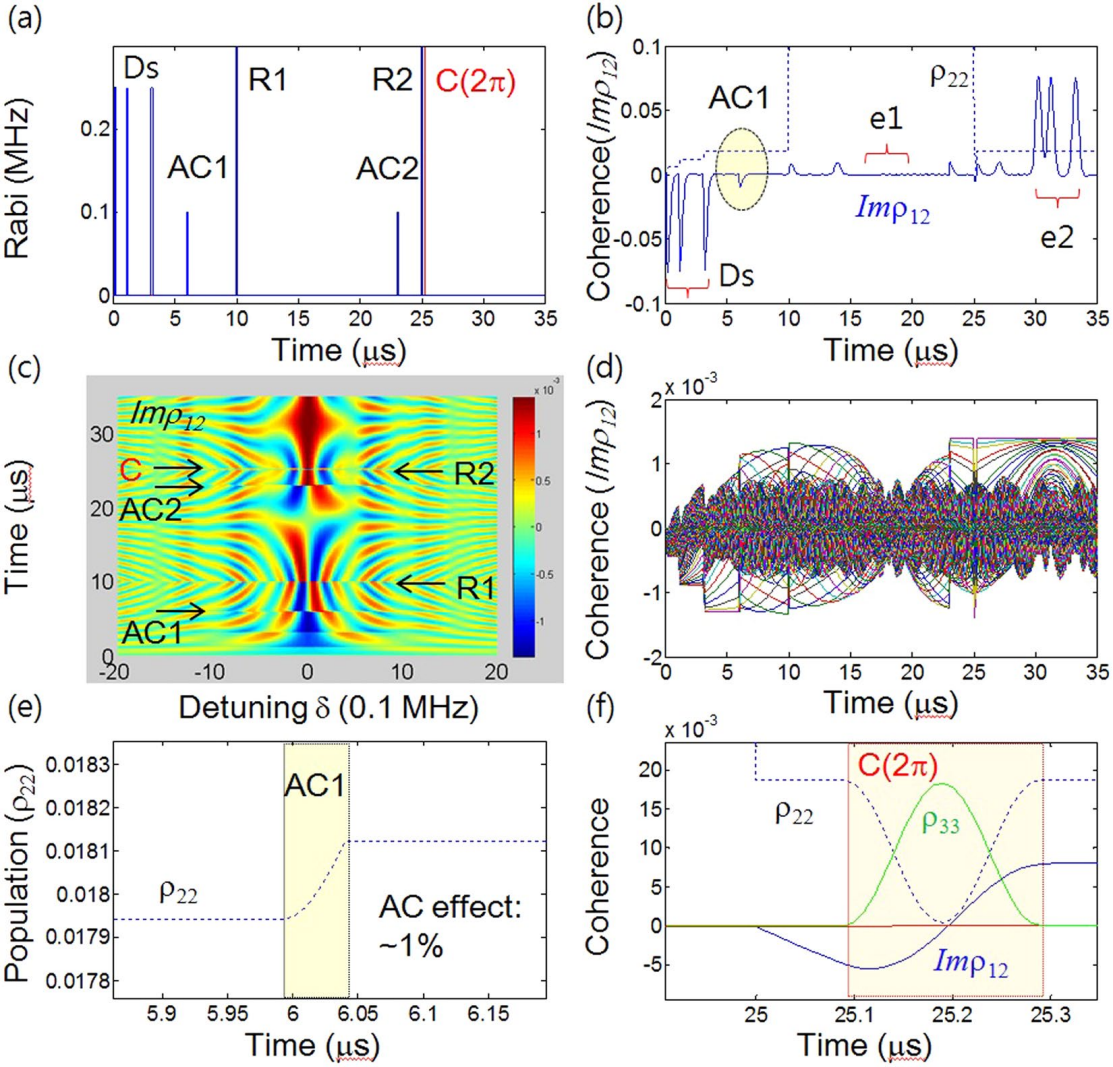
Controlled ac Stark echoes (CASE) in doubly rephased photon echoes for multiple weak data Ds. Three consecutive D pulses at 0.1,1.1, and 3.1 μs with the same area of π/20. Ω_AC1_ = Ω_AC2_ = 0.1 MHz. Φ_AC1_ = Φ_AC2_ = π/2. Δ_AC1_ = Δ_AC2_ = 5 MHz. Ω_R1_ = Ω_R2_ = 50 MHz. Ω_C_ = 5 MHz. All decay rates are zero. Optical inhomogeneous broadening is 1.7 MHz. (**a**) Pulse sequence. (**b**) Numerical results of (**a**). (**c**) 3D figure of (**b**). (**d**) Individual coherence evolution of (**b**). (**e**) ac Stark pulse-induced population change on ρ_22_. (**f**) Control Rabi flopping induced coherence inversion.

**Figure 6:**
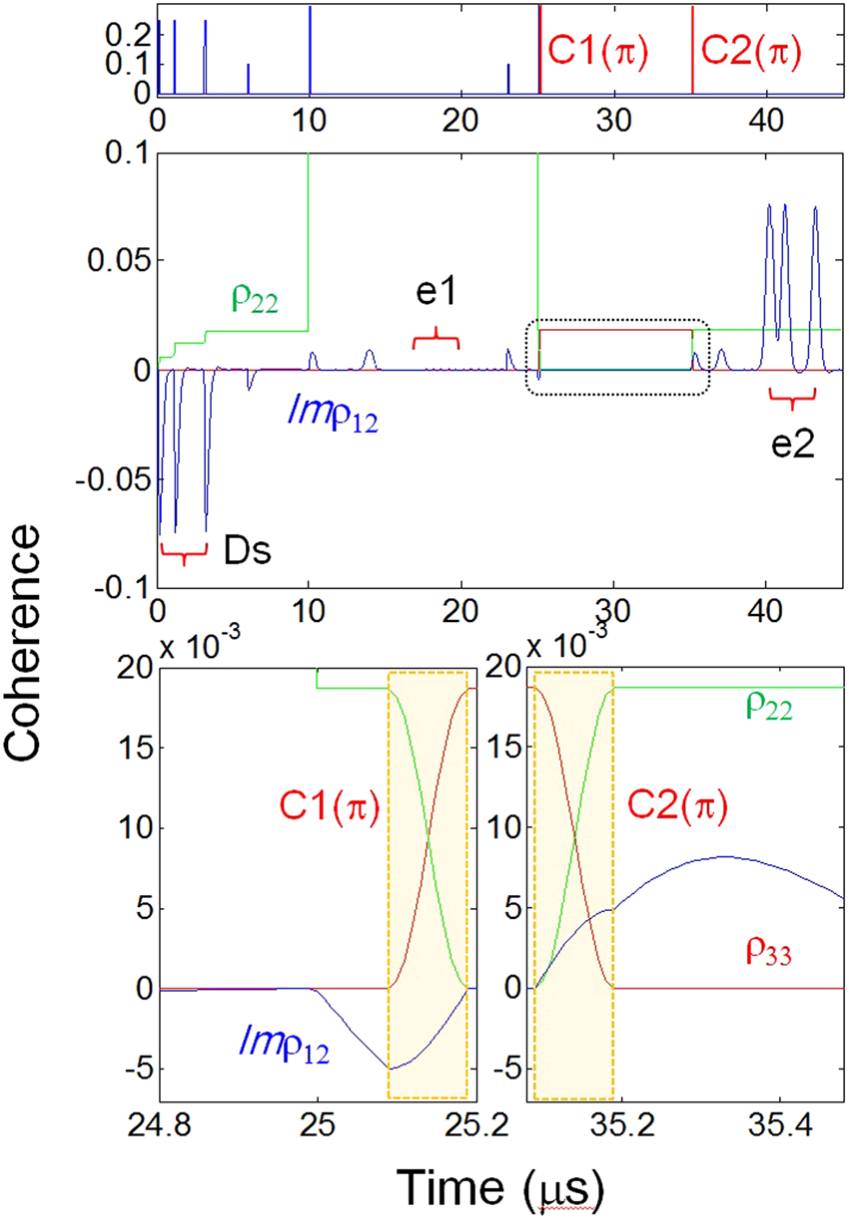
Storage time extended CASE. Top panel: Pulse sequence for multiple data storage. Middle panel: Storage time extension. Bottom panel: Coherence inversion (expansion of the dotted box in the middle panel). All parameters are the same as in Fig. 5 except for the splitting of C into C1 and C2.

Before proceeding toward the discussion of CASE, we first analyze on-demand atom phase control via ac Stark effect to erase the first echo (see Figs 1, 2 and 3). The basic physics of CCC is introduced in the Supplementary Information (Fig. S2)^17^, and the present quantum memory protocol CASE is discussed in Figs 4, 5 and 6, where the control Rabi pulse for CCC whose pulse area is 2π is resonant between the excited state $|2\rangle$ and an auxiliary ground state $|3\rangle$ (see Fig. 1a). For the discussion, we numerically solve nine time-dependent density matrix equations obtained via Liouville-von Neumann equations under rotating wave approximations for an inhomogeneously broadened Λ-type three-level system interacting with multiple optical pulses (see Methods)^27^. The CASE is an extended version of the ac Stark modulation^23^ to a double rephasing scheme combined with CCC, where double rephasing itself has a critical defect of absorptive coherence for the final echo resulting in its impracticality for quantum memories. The generalized Rabi frequency *Ω*′ by the ac Stark field with a detuning Δ_*AC*_ in a two-level system is given by $\Omega ^{\prime} =\sqrt{{{\rm{\Omega }}}_{AC}^{2}+{\Delta }_{AC}^{2}}$, where Ω_*AC*_ is the Rabi frequency of the ac Stark field. The question then becomes *what* Φ_*AC*_ *(ac Stark phase shift) is required to silence echo e1*.

To resolve this question, we start with a dressed state picture induced by the ac Stark field AC, as seen in Fig. 1a. In a two-level system composed of a ground state $|1\rangle \,({\omega }_{1})$ and an excited state $|2\rangle \,({\omega }_{2})$, the interaction Hamiltonian with an ac Stark field results in dressed states: $|2\pm \rangle \,({\omega }_{\pm })={\omega }_{b}+\frac{1}{2}{{\rm{\Delta }}}_{AC}^{^{\prime} }\pm \frac{1}{2}{\rm{\Omega }}^{\prime} \,$ ^28^. This optical system must be inhomogeneously broadened by Δ_*inh*_ to satisfy the photon echo condition. Thus, for the ac Stark field, the individual atom detuning is denoted by ${{\rm{\Delta }}}_{AC}^{^{\prime} }={{\rm{\Delta }}}_{AC}+{\delta }_{j}$, where *δ*_*j*_ is the detuning of the *j* ^*th*^ atom in Δ_*inh*_ from the line center. Because Δ_*inh*_ is symmetric for the resonance frequency, we treat the system as a collection of symmetrically detuned atom pairs ±*j* across the line center. The generalized Rabi frequency of AC is expressed by $\Omega ^{\prime} ={{\rm{\Delta }}}_{AC}^{^{\prime} }\sqrt{1+{(\frac{{{\rm{\Omega }}}_{AC}}{{{\rm{\Delta }}}_{AC}^{^{\prime} }})}^{2}}={{\rm{\Delta }}}_{AC}^{^{\prime} }(1+\frac{{{\rm{\Omega }}}_{AC}^{2}}{2{{\rm{\Delta }}}_{AC}^{^{\prime} }})$. In a bare state view for ${{\rm{\Delta }}}_{AC}\gg {{\rm{\Omega }}}_{AC};{{\rm{\Delta }}}_{inh}$, the ac Stark-induced frequency shift Δ_S_ from state $|2\rangle$ is ${\Delta }_{S}\simeq \frac{{{\rm{\Omega }}}_{AC}^{2}}{2{{\rm{\Delta }}}_{AC}}$, and the related frequency of $|2-\rangle$ is ${\omega }_{-}={\omega }_{b}+{{\rm{\Delta }}}_{S}$. Here our task is to minimize absorption, while maximizing dispersion (phase shift) by the ac Stark field for the effective phase shift. Any ac-Stark-caused absorption results in coherence dephasing, and thus degrades the echo efficiency. Hence, a weak Rabi frequency Ω_*AC*_ compared with the detuning Δ_*AC*_ is an essential condition (discussed in Section E).

### ac Stark modulation: echo erasing

Figure 1a,b are the energy level diagram and pulse sequence of the ac Stark modulations, respectively. Figure 1c,d are the corresponding results. Because the second echo e2 is the result of macroscopic rephaing of the first echo e1 by the second rephasing pulse R2, e2 must be affected by any macroscopic coherence burst of e1. This is why the first echo e1 must be erased (silent). To achieve the doubly rephased photon echo in Fig. 1b, there are two important tasks with regard to atom phase control. *First, echo e1 must be silent so that it does not affect echo e2. Second, echo e2 must be emissive if it is to be radiated out of the medium*. To begin with we analytically derive how D-excited atoms’ evolutions are described using time-dependent density matrix equations under rotating wave approximations. The key evolution parameter in coherent transients like photon echoes is the detuning δ_j_ in Δ_*inh*_:$\,{\rho }_{12}^{\pm j}({\rm{t}})={\rho }_{12}^{\pm j}(0){e}^{\pm i{\delta }_{j}t}$, where ${\rho }_{12}^{\pm j}$ is the density matrix element for the ±*j* ^*th*^ atoms denoting the coherence between states $|1\rangle$ and $|2\rangle$. In a Gaussian distributed ensemble, the coherence evolution of symmetrically detuned atom pairs at ±δ_j_ can be expressed by only the evolution term ${e}^{\pm i{\delta }_{j}t}$ for simplicity. Remembering that a π-rephasing pulse R reverses the phase evolution (${e}^{\pm i{\delta }_{j}T}\mathop{\to }\limits^{R(\pi )}{e}^{\mp i{\delta }_{j}T}$), the silent echo can be achieved by inserting either random phase turbulence or a controlled phase shift. In this Report, we use the controlled phase shift. Via the inserted ac Stark field (AC) in Fig. 1b, a phase shift ${{\rm{\Phi }}}_{AC}(\tau )$ is added to each D-excited atom coherence: ${e}^{\pm i{\delta }_{j}t}\to {e}^{\pm i{\delta }_{j}t+i{{\rm{\Phi }}}_{AC}(\tau )}$ & ${{\rm{\Phi }}}_{AC}(\tau )={{\rm{\Delta }}}_{S}\cdot \tau$. The sign of ${{\rm{\Phi }}}_{AC}(\tau )$ is predetermined by AC detuning, where ‘−’ (‘+’) represents a blue (red) detuning. This means that each interacting atom is simply frequency shifted by Δ_S_, and the phase evolution of each atom is accelerated (or decelerated) by the ac Stark-induced phase shift.

For direct numerical calculations without any assumption, however, density matrix solutions do not work for the dressed state picture or the ac Stark shift, unless an extra coherent probe field is added for an extended Raman system. The extended scheme is not for two-level system interactions anymore. Because the ac Stark effect simply adds a phase shift of ${{\rm{\Phi }}}_{AC}$ to each atom’s phase evolution as described above, we can still keep the original scheme by adapting the generalized frequency *Ω*′. This *Ω*′ treatment does not affect the result or violate the physics of ac Stark interactions, but does shorten phase shift time due to $\Omega ^{\prime} \gg {{\rm{\Delta }}}_{S}$. Another benefit of Ω′ treatment is that the ac Stark field-induced population change can be traced in real time. Although the ac Stark interaction assumes a negligibly small population change, it is good to check whether the ac Stark induced population change affects the echo efficiency.

Figure 1c shows the ac Stark-induced photon echo erasing for ${{\rm{\Phi }}}_{AC}=\pi /2$. Figure 1d shows coherence evolutions of all individual atoms in Fig. 1c. The phase addition of π/2 by AC to the system coherence ρ_12_(t) results in cancellation of coherence for the first echo e1. This actually effects coherence swapping between the real and imaginary parts of ρ_12_(t), where *Reρ*_12_ is zero for the first echo signal without AC (see the Supplementary Information Fig. S1(f)): ${e}^{\pm i{\delta }_{j}t}\to {e}^{\pm i({\delta }_{j}t+\pi /2)}$; $Re{\rho }_{12}\iff Im{\rho }_{12}$. Thus, *Imρ*_12_ (absorption) becomes zero at the e1 timing as shown in Fig. 1c,d. Here it should be noted that individual atom phase evolutions are not affected by the macroscopic coherence. Thus, the first task of silent echo e1 is achieved, with the condition of ${{\rm{\Phi }}}_{AC}=\pi /2$. In other words, with the unbalanced ac Stark field applied to the double rephasing scheme, the echoes can be dynamically erased in real time.

To understand the photon echo erasing mechanism by AC in Fig. 1, detailed analyses are performed in Fig. 2 for the same parameters as in Fig. 1 except for Ω_*AC*_. Figure 2a shows that the first echo (e1) amplitude degrades as Ω_*AC*_ increases for a fixed AC pulse duration τ up to ${{\rm{\Phi }}}_{AC}=\pi /2$, which is denoted by different colored curves:

${{\rm{\Phi }}}_{AC}=\frac{\pi }{5}(cyan);\frac{\pi }{4}(red);\frac{\pi }{3}(green);\frac{\pi }{2}(blue);\frac{2\pi }{3}(black);\frac{3\pi }{4}(magenta);\pi (dotted)$. To analyze the results in Fig. 2a a simple model is intuitively introduced, in which the e1 amplitude efficiency η is a cosine function of Φ_*AC*_:

1 $$\eta ({\rm{e}}1)\propto \,\cos \,{{\rm{\Phi }}}_{AC}.$$

Figure 2b shows the results of equation (1) applied to the Gaussian distributed ensemble, where the colored dots are for the e1 amplitude ratio to the maximum coherence of 0.5 in Fig. 2a. The solid curve is for overall η for all Gaussian distributed atoms, where the optical bandwidth is intentionally reduced by a factor of 2 (${{\rm{\Delta }}}_{inh}:1.7\,MHz\,\to 850\,kHz$) to test the bandwidth-dependent damping rate of η. As shown, all colored dots fit well with equation (1), except for the π pulse area indicated by the arrow. The damping of η is due to random phases among coherently excited atoms in Δ_inh_, such as in the free induction decay. Because the damping in η should be accelerated with bigger Δ_inh_, this discrepancy for ${{\rm{\Phi }}}_{AC}=\pi$ is quite reasonable. For ${{\rm{\Delta }}}_{inh}=1.7\,MHz$, the data (${{\rm{\Phi }}}_{AC}=\pi$) fits well with equation (1) (not shown). From Fig. 2b, we induce the following conclusion: The general condition of the echo erasing (silencing) by the ac Stark field is

2 $${{\rm{\Phi }}}_{AC}=(2n-1)\pi /2,$$

where the damping rate is accelerated by the ensemble broadening Δ_inh_. This intuitive model of equation (1) has also been confirmed experimentally^29^: ${I}_{e1}\propto \frac{1}{2}(1+\,\cos \,2{{\rm{\Phi }}}_{AC})\,$. Thus, the echo efficiency can be accurately controlled simply by adjusting the pulse area AC. Moreover, η is insensitive to Δ_inh_ for ${{\rm{\Phi }}}_{AC} \sim \pi /2$. The dashed curve is for the resonant atoms (δ_j_ = 0; ${{\rm{\Delta }}}_{AC}^{^{\prime} }={{\rm{\Delta }}}_{AC}$) and is provided as a reference.

### Analytic expression for ac Stark echo: Part I

Figure 3a,b, respectively, show two different cases of unbalanced (asymmetric) and balanced (symmetric) ac Stark fields applied to CASE. If AC2 is turned on before (after) e1 and Ω_AC2_ = Ω_AC1_, then it is called ‘balanced’ (‘unbalanced’). Figure 3c,d are the corresponding results. Figure 3e,f show the details of Fig. 3c,d, respectively, for all individual atoms. As shown in Fig. 3c,e, the unbalanced CASE results in the erasing (silence) of e1 (see the blue center curve). However, the resultant echo e2 is absorptive (notice that e2 has the same sign as D). To understand the physics of atom phase evolution, we can treat the interaction system analytically as follows. For the pulse sequence of D → ACI → RI in Fig. 3a the corresponding phase evolutions of the D-excited atoms are expressed by:

3 $${e}^{\pm i{\delta }_{j}t}\mathop{\longrightarrow }\limits^{AC1}{e}^{\pm i{\delta }_{j}t+i{{\rm{\Phi }}}_{AC1}}\mathop{\longrightarrow }\limits^{R1}{e}^{\mp i{\delta }_{j}T-i{{\rm{\Phi }}}_{AC1}\pm i{\delta }_{j}t^{\prime} },$$

where $t^{\prime} =t-T$, $T={t}_{R1}-{t}_{D}\,(=6\,\mu s)$. The *t*_*k*_ stands for the arrival time of pulse *k*, and ‘*t*′’ is the time after t_R1_. Here, in this analytic expression, we use *a priori* knowledge of a π phase shift by R1 (π pulse area) in a two-level system: ${\rm{\rho }}({\rm{t}})\mathop{\longrightarrow }\limits^{R1}\rho {(t)}^{\ast }$. As discussed in Figs 1 and 2, for ${{\rm{\Phi }}}_{AC1}=\frac{\pi }{2}$, all *Im*ρ_12_ values become zero at $t^{\prime} =T\,({\rm{t}}=2{\rm{T}})$ for the e1 timing: ${e}^{\mp i{\delta }_{j}T-i{{\rm{\Phi }}}_{AC1}\pm i{\delta }_{j}t^{\prime} }\to {e}^{-i\pi /2}=-i$. It should be noted that the imaginary value (−*i*) in this phase evolution notation is actually for *Re*ρ, while the real value is for *Im*ρ, according to the definition ${\rho }_{12}^{\pm j}({\rm{t}})={\rho }_{12}^{\pm j}(0){e}^{\pm i{\delta }_{j}}$. Remembering the π/2 phase shift between *Im*ρ_12_ and *Re*ρ_12_, the π/2 ac Stark field functions are to swap these two quantities, resulting in *Im*ρ_12_ = 0 while *Re*ρ_12_ is a maximum, as discussed in Fig. 1 (see the swapping of blue and red curves across AC2 in Fig. 3c).

The phase added by AC1, however, can be completely compensated for by AC2, making the final echo e2 unchanged by AC1 via erasing e1. For ${{\rm{\Phi }}}_{AC2}={{\rm{\Phi }}}_{AC1}$, the atom phase evolution by the pulse sequence of AC2 → R2 after R1 is expressed by:

4 $${e}^{\pm i{\delta }_{j}(t^{\prime} -T)-i{{\rm{\Phi }}}_{AC1}}\mathop{\to }\limits^{AC2}{e}^{\pm i{\delta }_{j}(t^{\prime} -T)-i{{\rm{\Phi }}}_{AC1}+i{{\rm{\Phi }}}_{AC2}}\mathop{\to }\limits^{R2}{e}^{\mp i{\delta }_{j}(T+T^{\prime} -T)\pm i{\delta }_{j}t^{\prime\prime} }={e}^{\pm i{\delta }_{j}(t^{\prime\prime} -T^{\prime} )},$$

where $T^{\prime} ={t}_{R2}-{t}_{e1}\,(=4\,\mu s)$ and $t^{\prime\prime} =t-(T^{\prime} +2T)$. The ‘*t*″’ is the time after t_R2_. At $t^{\prime\prime} =T^{\prime} \,(or\,t=2(T^{\prime} +T))$ for the e2 timing, the echo e2 is generated as shown in Fig. 3c: ${e}^{\pm i{\delta }_{j}(t^{\prime\prime} -T^{\prime} )}\to 1$ (Max Imρ_12_). However, the echo e2 is absorptive, and cannot be radiated out of the medium. On the other hand, turning on AC2 (${{\rm{\Phi }}}_{AC2}={{\rm{\Phi }}}_{AC1}$) before e1 in Fig. 3b has no effect, as shown in Fig. 3d: ${e}^{\pm i{\delta }_{j}t+i{{\rm{\Phi }}}_{AC1}}\mathop{\to }\limits^{R1}{e}^{\mp i{\delta }_{j}T-i{{\rm{\Phi }}}_{AC1}}\mathop{\to }\limits^{AC2}{e}^{\mp i{\delta }_{j}T-i{{\rm{\Phi }}}_{AC1}+i{{\rm{\Phi }}}_{AC2}}={e}^{\mp i{\delta }_{j}(t^{\prime} -T)};$ $t^{\prime} =t-T$. The balanced ac Stark effect in Fig. 3d is the same as the bare double rephasing as shown in the Supplementary Information Fig. S1. This is due to the complete phase cancellation between AC1 and AC2 before e1 formation. Thus, the on-demand phase manipulations for echo erasing are achieved by using the ac Stark fields.

### Analytic expression for ac Stark echo: Part II

The absorptive echo e2 in Fig. 3c can also be driven analytically without using phase evolution terms. Using the phase shift relationship of ${\rho }_{12}\mathop{\longrightarrow }\limits^{R(\pi )}{\rho }_{12}^{\ast }$ and $Im{\rho }_{12}\mathop{\longleftrightarrow }\limits^{AC(\frac{\pi }{2})}Re{\rho }_{12}$ as discussed in Figs 1 and 2, the atom coherence change for the pulse sequence of ${\rm{D}}\to {\rm{AC}}1\to {\rm{R}}1\to {\rm{AC}}2\to {\rm{R}}2$ in Fig. 3a is expressed by:

5 $$Im{\rho }_{12}:i{\rm{\rho }}({t}_{D})\,\mathop{\longrightarrow }\limits^{AC1(\frac{\pi }{2})}r{\rm{\rho }}({t}_{AC1})\mathop{\longrightarrow }\limits^{R1(\pi )}{[r{\rm{\rho }}({t}_{R1})]}^{\ast }\mathop{\longrightarrow }\limits^{AC2(\frac{\pi }{2})}{[i{\rm{\rho }}({t}_{AC2})]}^{\ast }\mathop{\longrightarrow }\limits^{R2(\pi )}i{\rm{\rho }}({t}_{R2}),$$

6 $$Re{\rho }_{12}:r{\rm{\rho }}({t}_{D})\mathop{\longrightarrow }\limits^{AC1(\frac{\pi }{2})}i{\rm{\rho }}({t}_{AC1})\mathop{\longrightarrow }\limits^{R1(\pi )}{[i{\rm{\rho }}({t}_{R1})]}^{\ast }\mathop{\longrightarrow }\limits^{AC1(\frac{\pi }{2})}{[r{\rm{\rho }}({t}_{AC2})]}^{\ast }\mathop{\longrightarrow }\limits^{R2(\pi )}r{\rm{\rho }}({t}_{R2}).$$

Here the *t*_*k*_ stands for the time right after the pulse *k*. Obviously the final echo e2 (last term) in equations (5) and (6) has the same form as the initial D-excited coherence (first term), resulting in an absorptive echo. After R1, there is no *i*ρ component in *Im*ρ_12_ due to the π/2−AC1 in equation (5), which represents the silent (erased) echo e1 as discussed in Figs 1, 2 and 3.

### Controlled ac Stark echoes: CASE

To convert the absorptive echo e2 into an emissive one, the present quantum memory protocol CASE is introduced in Fig. 4. As shown in Fig. 4a, a control 2π pulse C is added after the second rephasing pulse R2 (but before e2), where the function of the control pulse is to induce coherence inversion via an optical Rabi flopping between the excited state $|2\rangle$ and an auxiliary state $|3\rangle$ (see Fig. 1a). The mechanism of the control pulse has been intensively studied under the name of CDR^14–19^, where the control pulse inverts the system coherence:${{\rm{\rho }}}_{12}({t}_{R2})\mathop{\longrightarrow }\limits^{C(2\pi )}-{{\rm{\rho }}}_{12}({t}_{C})$. Thus, the absorptive echo (e2) in Fig. 3c becomes emissive, as shown in Fig. 4b. Echo e2 has exactly the same form as the original two-pulse photon echo, but without the population inversion as a direct result of double rephasing (see also Supplementary Information Fig. S3). The first echo e1 is completely erased by AC1 $({{\rm{\Phi }}}_{AC1}=\pi /2)$ due to $Im{\rho }_{12}\leftrightarrow Re{\rho }_{12}$ as discussed in Figs 1, 2 and 3. The details of coherence inversion are shown in Fig. 4c for a detuned individual atom at δ_j_ = 150 kHz in Fig. 4b. By the control pulse C, the signs of both *Re*ρ_12_ and *Im*ρ_12_ are exactly reversed, which is known as CCC (see also the Supplementary Information Fig. S3(d)).

For the final e2 in CASE of Fig. 4, the system population is nearly the same as that for data absorption owing to double rephasing (will be discussed in Section E), i.e., there is no population inversion. Due to the large detuning of ac Stark fields the population redistribution via AC is negligible. Any absorption (population) change by the ac Stark fields, however, should affect the echo efficiency due to the phase turbulence. The comparison between the ac Stark induced population change and phase shift is discussed in Section E. By splitting 2π-C into two π-Cs, a near perfect ultralong quantum memory protocol can be achieved, which is discussed in Section F.

### CASE: Weak-field limit

Figure 5 shows multiple bit quantum memory of CASE for a weak field limit, whose binary code of the data Ds is 1101. Each data pulse is extremely weak, where the pulse area is ${{\rm{\Phi }}}_{D}=\frac{\pi }{20}\,\,$for each pulse, corresponding to a 250 kHz Rabi frequency with a fixed pulse duration of 0.1 μs. Other parameters are noted in the figure caption. Figure 5a is the pulse sequence, and Fig. 5b is the corresponding numerical results. As shown in Fig. 5b, the consecutive data pulses are retrieved in the same order as the data pulse sequence owing to the double rephasing mechanism. Compared to the reversed order in two-pulse photon echoes such as CRIB^1–5^ and AFC^6–9^, this same order sequence in multiple-bit storage offers an important benefit for direct information processing without additional conversion processes. The dotted line in Fig. 5b represents the population (ρ_22_) evolution in the excited state $|2\rangle$, where the population difference between the regions right after Ds and after R2 is negligibly small at ~2% (discussed in Fig. 5e). Figure 5c,d show the coherence evolutions of all individual atoms from Fig. 5b. As shown in Fig. 5c, the ac Stark pulse AC1 adds a particular phase of π/2 to each atom, resulting in a silent echo e1 as discussed in Figs 1, 2, 3 and 4. As discussed in Figs 3 and 4, the phase shift due to AC1 is completely cancelled by AC2 owing to the rephasing process by R1, resulting in e2 remaining intact.

Figure 5e is the expansion of the dotted circle in Fig. 5b for the population (ρ_22_) change of 1% by AC1. Due to the weak Rabi frequency Ω_AC_, the population change (absorption) is minimized, while the π/2 phase shift (dispersion) can be obtained. Balancing the absorption and dispersion is the key role of the ac Stark field in CASE, where any population change by the ac Stark fields must affect e2. Thus, the ac Stark field-induced coherence dephasing due to absorption must be carefully controlled for it to be minimized, especially for single photon-based quantum memory applications. Figure 5f shows coherence inversion by the 2π-C control pulse via Rabi flopping between states $|2\rangle$ and an auxiliary ground (spin) state $|3\rangle$. Owing to the (single) Rabi flopping there is no population change in ρ_22_ before and after C. However, the system coherence ρ_12_ is inverted as mentioned in Fig. 4c. In the middle of C, the population ρ_22_ in the excited state $|2\rangle$ is completely transferred into the auxiliary spin state $|3\rangle$ (${{\rm{\rho }}}_{22}\mathop{\longrightarrow }\limits^{C(\pi )}{\rho }_{33}$), and the optical coherence ρ_12_ becomes zero: ${\rho }_{12}\mathop{\longrightarrow }\limits^{C(\pi )}0$. Thus, not only optical dephasing, but also optical evolution stops at this mid-point (discussed in Section F with regard to storage time extension). These are the novel characteristics of CCC, distinguishing a three-level system from the two-level system. The physics of coherence inversion in Fig. 5f originates from two-photon coherence in a resonant Raman system^30, 31^. In such a two-photon coherence system, the harmonic oscillation period for the coherence is based on a 4π modulus (see also the Supplementary Information Fig. S3(e) and (f))^17, 30, 31^. Here in CCC, only half of the complete process is performed by one leg (C) of the Raman pulse pair for ρ_22_, resulting in a half phase shift, π.

Another issue to discuss is the imperfect rephasing by R due to the atom detuning δ_j_ in Δ_inh_, resulting in imbalanced population swapping. To minimize the Δ_inh_ effect on imbalanced swapping a bigger Rabi frequency Ω_R_ must be used. In Figs 5 and 6, Ω_R_ is increased by one order from 5 MHz to 50 MHz. Thus, the spectral channel bandwidth for signal multiplexing in CASE may be limited by the rephasing Ω_R_ rather than the ac Stark detuning Δ_AC_. Considering the ~10 GHz Δ_inh_ in rare-earth doped solids, the possible number of spectral channels for quantum memory multiplexing reaches a few hundreds, though. To avoid rephasing-induced unwanted quantum noises, spectral preparation for an anti-hole in a wide spectral pit may be needed for each spectral channel^32^. Considering three hyperfine states in the ground level of rare earth-doped solids, this spectral preparation, however, is not challenging^30–32^.

### Backward CASE: Near perfect, ultralong quantum memory

In Fig. 6 we discuss near perfect ultralong CASE by replacing the single 2π-C in Fig. 5 by two time-delayed π-C pulses, where the pulse sequence is shown in the top panel. All other parameters are the same as in Fig. 5. The corresponding results are shown in the middle panel, in which the storage time is extended by the delay $T^{\prime} \,({t}_{C2}-{t}_{C1})$ of C2 assuming no spin dephasing. In an actual solid medium, however, the spin dephasing (due to spin inhomogeneous broadening) is severe, as demonstrated in a Pr^3+^-ion doped Y_2_SiO_5_ crystal^8, 31^. The spin dephasing can be minimized by simply applying Zeeman fields, so that *T*′ can be as long as spin phase decay time^33^. As discussed in Fig. 5f, the π-pulse of C1 stops all-optical dephasing process until C2 is turned on. As shown in the bottom panel, no optical coherence change happens between C1 and C2 (see also the dotted box in the middle panel). Even though the dynamic decoupling (DD) technique^26^ is limited to quantum information processing with many gate operations, DD can be used for quantum repeaters, where *T*′ with DD can be extended up to the spin population decay time, which is in the order of a minute in Pr:YSO^26^.

For near unity retrieval efficiency a backward echo scheme using the control pulse pair C1 and C2 has been suggested^1, 2, 14, 16^ and experimentally demonstrated^31^ for a single rephasing scheme. Unlike rephasing-based backward echo techniques^1, 2, 10, 11^, the present backward CASE relies on Cs via nonlinear quantum optics^31^. The phase matching conditions for the backward CASE are as follows:

7 $${\vec{k}}_{e2}={\vec{k}}_{D}-{\vec{k}}_{C1}+{\vec{k}}_{C2},$$

8 $${\omega }_{e2}={\omega }_{D}-{\omega }_{C1}+{\omega }_{C2},$$

where ${\vec{k}}_{j}$ (${\omega }_{j}$) is the wave vector (angular frequency) of the pulse j. From equations (7) and (8) the identical wavelength condition (${\omega }_{e2}={\omega }_{D}$) is confirmed, but the propagation direction between D and e2 is not exactly opposite: ${\vec{k}}_{e2}\ne -{\vec{k}}_{D}$. This propagation deviation, however, is within the interaction volume inside the medium owing to ~10^−8^ in the frequency ratio between them, resulting in a ~0.1 mrad deviation^31^. Considering both ~mm-length optical medium with ~0.1 mm-wide interaction cross section, equation (7) is satisfied for potentially any angles. This fact has already been demonstrated in many (rare-earth) nonlinear optics experiments and has drawn much attention recently for its use in high-etendue medical imaging^34^. If this mechanism were rephasing-based, i.e., ${\vec{k}}_{e}=2{\vec{k}}_{R(C)}-{\vec{k}}_{D}$ as in refs 1 and 10. the phase mismatching would never allow echo formation since $(|{k}_{e}|-|{k}_{D}|)L\gg \pi$, where L is the medium length. Thus, the present backward CASE has the practical benefit of spatial multiplexing for a wide angle in multi-spectral channels.

Due to the fundamental limitation of the density matrix equations applicable to only the time domain, Maxwell-Bloch (MB) equations must be used for the retrieval efficiency calculations, which is beyond the scope of the present Report. Thus, we have separately provided the retrieval efficiency of backward CDR echoes in a double rephasing scheme in ref. 19. Because the ac Stark field has nothing to do with the retrieval efficiency based on equations (7) and (8) due to the complete phase cancellation before R2, the retrieval efficiency η of the present backward CASE is given by ref. 19:

9 $${\rm{\eta }}={(1-{e}^{-\alpha L})}^{2},$$

where *αL* represents the optical depth of the medium. Therefore in an optically dense medium $(\alpha L\gg 1)$ the retrieval efficiency of backward CASE is almost unity. Here, an optically dense medium is also necessary for all ensemble-based quantum memory techniques for complete data transfer via full absorption. Although the echo efficiency has nothing to do with the fidelity, the near perfect retrieval efficiency provides a critical condition for fault-tolerant quantum computing with thousands of gate operations^20^ and loophole-free quantum communications via quantum interface^21^.

## Conclusion

In summary a new quantum memory protocol of controlled ac Stark echoes (CASE) was presented in a double rephasing photon echo scheme with an unbalanced ac Stark pulse pair and controlled Rabi pulse(s), where the control Rabi pulse(s) converts the absorptive echo into an emissive one. The function of ac Stark pulse is to erase (silence) the first echo so that the first echo does not affect the final echo. An exact equation of ac Stark pulse area for erasing the first echo was induced. Using time-dependent density matrix equations, multiple-bit CASE was successfully demonstrated in a weak field limit without any approximations. The multiple-bit retrieval order was the same as for the data bit sequence, providing an essential benefit of CASE for direct quantum information processing without additional conversion steps. In addition to full numerical calculations, analytical evaluations were also given for the same results to provide conceptual understanding. For an extended version of CASE, storage-time extension was discussed by splitting the control Rabi pulse into two time-delayed π-control pulses. In this storage-time extended version, a near unity retrieval efficiency was obtained in an optically dense medium via the backward CASE scheme, where wide angle flexibility offers the practical benefits of spatial multiplexing for spectral channels^35^. Thus, combined with its intrinsic property of spectral multiplexing, the present quantum memory protocol of CASE can be applied to various multi-mode quantum memory applications utilizing all spectral, temporal, and spatial domains^36^. The near perfect retrieval efficiency of the present scheme should contribute to fault-tolerant quantum computing and loophole-free quantum communications via a quantum interface.

## Methods

For the numerical analyses, the time-dependent density matrix equations, ${\dot{\rho }}_{ij}\,(=-\frac{i}{\hslash }[H,\rho ]+decay\,terms)$, are obtained by solving the time-dependent Schrodinger equations under rotating wave approximations, ${\rm{i}}\hslash |\dot{{\rm{\Psi }}}\rangle =H|{\rm{\Psi }}\rangle$ ($H\,is\,the\,interaction\,Hamiltonian;\,\rho =|{\rm{\Psi }}\rangle \langle {\rm{\Psi }}|$)^27^, and they were numerically solved without approximations. The following equations are for the coherence terms of ${\dot{\rho }}_{ij}\,\,$in a lambda-type, three-level system interacting with several resonant/off-resonant optical fields:

10 $$\frac{d{\rho }_{12}}{dt}=-\frac{i}{2}{{\rm{\Omega }}}_{1}({\rho }_{11}-{\rho }_{22})-\frac{i}{2}{{\rm{\Omega }}}_{2}{\rho }_{13}-i{\delta }_{1}{\rho }_{12}-{\gamma }_{12}{\rho }_{12},$$

11 $$\frac{d{\rho }_{13}}{dt}=-\frac{i}{2}{{\rm{\Omega }}}_{2}{\rho }_{12}+\frac{i}{2}{{\rm{\Omega }}}_{1}{\rho }_{23}-i({\delta }_{1}-{\delta }_{2}){\rho }_{13}-{\gamma }_{13}{\rho }_{13},$$

12 $$\frac{d{\rho }_{23}}{dt}=-\frac{i}{2}{{\rm{\Omega }}}_{2}({\rho }_{22}-{\rho }_{33})+\frac{i}{2}{{\rm{\Omega }}}_{1}{\rho }_{21}+i{\delta }_{2}{\rho }_{23}-{\gamma }_{23}{\rho }_{23},$$

where the interaction Hamiltonian matrix H is

13 $${\rm{H}}=-\frac{\hslash }{2}[\begin{array}{ccc}-2{\delta }_{1} & {{\rm{\Omega }}}_{1} & 0\\ {{\rm{\Omega }}}_{1} & -2{\delta }_{2} & {{\rm{\Omega }}}_{2}\\ 0 & {{\rm{\Omega }}}_{2} & 0\end{array}].$$

Here Ω_1_ is the Rabi frequency of the optical field between the ground state $|1\rangle$ and the excited state $|2\rangle$, Ω_2_ is the Rabi frequency of the control field between the auxiliary ground state $|3\rangle$ and the excited state $|2\rangle$, and δ_1_ (δ_2_) is the atom detuning from the field Ω_1_ (Ω_2_). For visualization purpose and simplification, all decay terms are neglected. To satisfy the ensemble-based quantum memory protocols particularly photon echoes, the interaction medium must be inhomogeneously broadened by Δ_inh_, where Δ_inh_ is Gaussian distributed. For this, we used a practical value of ${{\rm{\Delta }}}_{inh}=1.7\,{\rm{MHz}}$ for the anti-hole in a wide spectral pit. To minimize the population swapping imbalance via rephasing pulse Ω_R_ due to atom inhomogeneity Δ_inh_, we used a bigger Rabi frequency of ${{\rm{\Omega }}}_{{\rm{R}}}=50\,\mathrm{MHz}$ for ultraweak data pulses in Figs 5 and 6. The ac Stark fields also may deteriorate system coherence via unwanted absorption. To limit the ac Stark-induced absorption change at ~1%, we used the following numbers: ${{\rm{\Omega }}}_{{\rm{AC}}}=0.1\,\mathrm{MHz};{{\rm{\Delta }}}_{{\rm{AC}}}=5\,\mathrm{MHz}$. In these calculations, all frequency parameters are multiplied by 2π. Ultraweak consecutive data pulses at ${{\rm{\Omega }}}_{D}=\frac{\pi }{20}$ each were used for practical CASE to reveal any coherence noise due to strong classical pulses of the ac Stark AC, rephasing R, and control C. The time step for the density matrix calculations was set to 0.01 μs. The data, rephasing, and ac Stark pulse durations were each set to 0.1 μs. The optical inhomogeneous width Δ_inh_ was divided into 401 groups at a step of 10 kHz to denote the detuning δ_j_ for each atom group. For the plots in all figures, all atom groups were calculated independently, and then summed according to δ_j_-dependent weights in the Gaussian distribution. To overcome the limitation of density matrix-based numerical calculations for ac Stark shift, we use the generalized Rabi frequency (Ω′) as the ac Stark phase shift (Δ_*S*_): ${{\rm{\Phi }}}_{AC}(\tau )={{\rm{\Delta }}}_{S}\tau \to {\rm{\Omega }}^{\prime} \tau \,$. In this Ω′ treatment, nothing changes except that the phase-shift time is shortened without violating the physics. Moreover, the ac Stark-affected population change can be seen for the effect of echo efficiency. Here any population change should affect phase shift. For comparison with the Ω′-based shortened time, a test version of the ac Stark effect in a Raman system was shown in Supplementary Information Fig. S4, where the phase shift of ${{\rm{\Phi }}}_{AC}(\tau )$ for ρ_12_ was inevitably added in the middle of processing. For all figures, Δ_S_ is replaced by Ω′.

## Electronic supplementary material

supplementary information
