# Supplementary material for: A controlled ac Stark echo for quantum memories
Source: Sci Rep. 2017 Aug 9;7:7655. doi: 10.1038/s41598-017-08051-5 (PMC5550504; doi:10.1038/s41598-017-08051-5)
Supplement: Supplementary file 1 — supplementary information [file 41598_2017_8051_MOESM1_ESM.pdf]

Supplementary Information for

A controlled ac Stark echo for quantum memories

Byoung S. Ham

Although conventional photon echoes were intensively studied in the 1980s and 1990s for all-optical information processing, it eventually failed to be practical for most applications due to low retrieval efficiency and cryogenic temperature operations. In the 2000s, however, the photon echo has been reborn as a quantum memory protocol with intrinsic benefits of wide bandwidth, multimode storage, and all-optical access capabilities. Other ensemble-based quantum memory techniques such as cavity-QED, off-resonant Raman echoes, and ultraslow light are basically single mode operations with a limited bandwidth, which thus cannot be practical for mass data processing.

In photon echo-based quantum memories a quantum coherence control of transient atoms plays a key role. A common misunderstanding of photon echoes for coherence manipulations of an ensemble stems from the confusion between 'rephasing' and 'quantum coherence control.' The 'rephasing' process of an ensemble is the key mechanism for the reversible coherence evolutions resulting in an echo signal, where population swapping between ground and excited states by an optical π pulse induces a π phase shift to the ensemble. Therefore, the absorptive coherence excited by a data pulse turns out to be emissive for the echo, where only emissive coherence releases a photon echo signal. Therefore, photon echoes must be prohibited in a double rephasing scheme only due to the absorptive coherence of the echo signal via a double π phase shift.

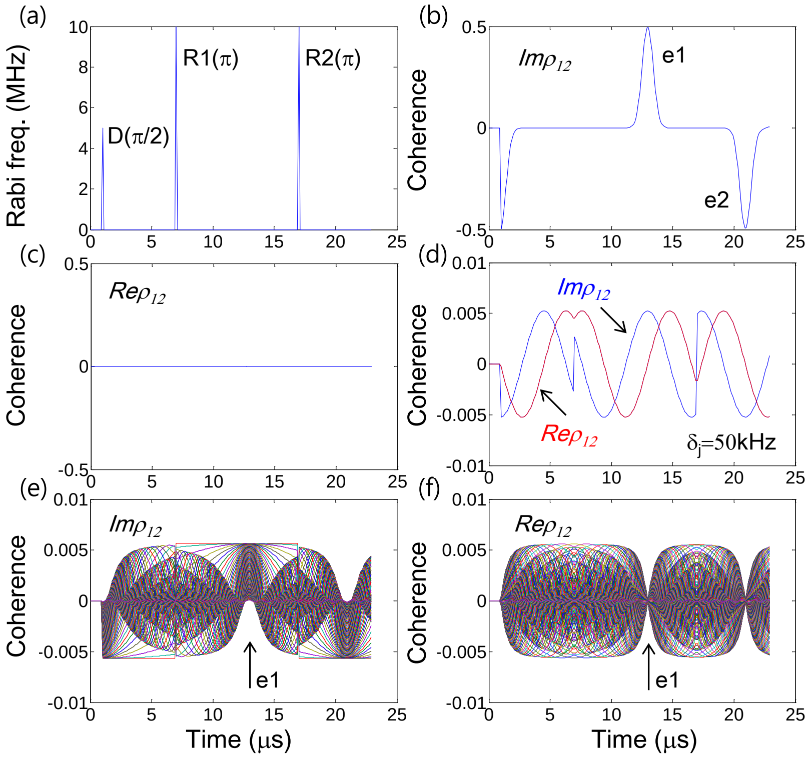

Figure S1. Doubly rephased photon echoes. (a) Pulse sequence: Data D, Rephasing R1, and Rephasing R2. (b)-(f) Numerical calculations for optical coherence based on time-dependent density matrix equations. (b) and (c) are for sum of all atoms, where (e) and (f) are corresponding all individual atoms. All other parameters are the same as in Fig. 1.

Figure S1 shows numerical simulations of double rephasing photon echoes. Figure S1(a) is the pulse sequence, and Figs. S1(b)~(f) are the corresponding results. The double rephasing photon echo scheme inherently provides two echoes e1 and e2, and the final echo e2 is under no population inversion. Because echo e2 is the rephasing of echo e1 by R2, echo e2 must be absorptive as shown in Fig. S1(b). The rephasing pulse

induces a π–phase shift to the coherence via a population swapping between two resonant states. Thus, odd numbered rephasing pulse scheme always induces an emissive photon echo under population inversion, while even numbered rephasing pulse scheme changes nothing resulting in an absorptive echo under no population inversion. Here, the second echo e2 must be affected by e1 if there is an echo signal generation through the medium. Thus, the echo e1 must be erased or silenced not to affect the final echo e2. Erasing (silencing) e1, however, does not affect individual coherence evolutions for e2. The first trial of silencing echo e1 was obtained by using a backward propagation scheme with R1 and R2 [10], where the propagation direction of the echo e2 becomes forward. Later other techniques followed [11-13,23]. Although the echo e2 detection is prohibited by the absorptive coherence, echoes have been actually observed in the double rephasing schemes [10-13,23] due to imperfect rephasing-caused coherence leakage by a Gaussian light pulse [22].

The physics of 'quantum coherence control' has been studied over decades in a three-level system for light-matter interactions, such as in electromagnetically induced transparency (EIT) and resonant Raman echoes. Like phase invariant light-matter interactions in a two-level system by a 2π pulse, the double rephasing process composed of consecutive two π pulses is also phase invariant to the ensemble coherence. However, a 2π control pulse resonant between the excited state (or the ground state) of the photon echo scheme and an auxiliary third state inverts the ensemble coherence [1,2,14-19,30]. In other words, quantum coherence control via Rabi flopping to a third state can convert the absorptive echo into emissive one. This kind of phase inversion control can never be achieved in a two-level system. Thus, the novelty of the present paper is to give a new protocol of a near perfect, storage time-extended quantum memory by using 1. ac Stark effects for silencing the first echo and 2. controlled coherence conversion (CCC) for atom phase control in the double rephasing photon echo scheme. The details of how the atom coherence excited by a data pulse can be manipulated according to light-matter interactions are presented in the following Fig. S2.

Figure S2(a) shows a three-level nonlinear optical medium interacting with two resonant optical pulses, where D stands for a data or rephasing pulse in a conventional photon echo scheme, and C stands for a control pulse in CCC for coherent atom phase control. Here, we assume that all decay rates among states $|1\rangle$, $|2\rangle$, and $|3\rangle$ are zero for simplicity. The light-matter interactions are numerically calculated by using time-dependent density matrix equations under rotating wave approximations as given in Methods. As shown in Fig. S2(b), the control pulse C whose pulse area is 2π induces phase inversion to all atom groups. The medium is inhomogeneously broadened whose bandwidth (FWHM) is 1.7 MHz. Figure S2(c) visualizes the phase inversion for all atoms (see the color swapping across the pulse C at t=2.0 μs). Here the phase inversion by the 2π–C is for both imaginary ($Im\rho_{12}$) and real ($Re\rho_{12}$) parts as shown in Fig. S2(d). Unlike rephasing process in photon echoes ($\rho \rightarrow \rho^*$; see the arrow for the 2π–D in Fig. S2(d)), the function of the 2π–C is the coherence inversion: $\rho \rightarrow -\rho$.

Detailed explanations of the atom coherence control are given in Figs. S2(e) and (f). The longer pulse usage in Fig. S2(e) is to show different functions of D and C, resulting in different coherence oscillation periods for the same pulse area. In a two-level system composed of states $|1\rangle$ and $|2\rangle$, Rabi oscillation induces coherence oscillation in the same pace (see the green (ρ_{22}) for population and blue ($Im\rho_{12}$) for coherence in the region of D pulse). On the contrary, the control pulse C induces half slow coherence oscillation ($Im\rho_{12}$) compared with that of ρ_{22} in the region of C pulse). This is due to the two-photon coherence ρ_{13} (dotted curve) between states $|1\rangle$ and $|3\rangle$ only via the population transfer by C, where ρ_{13} has a twice longer oscillation period. Because density matrix elements are coupled with one another, the optical coherence ρ_{12} changes according to ρ_{13} under the action of the control pulse C (see Methods). Figure S2(f) clearly visualizes this C pulse-induced distinct effects. As a result, nothing happens on ρ_{13} if there is no population in the excited state $|2\rangle$ (not shown). Thus, as shown in Fig. S2(b), the 2π–C pulse induces coherence inversion on the system coherence ρ_{12}. Figures S2(g) and (h) represent a phase controlled photon echo by using 2π–C pulse, where the echo turns out to be absorptive. This is how CCC plays to convert the absorptive echo into an emission one in a double rephasing scheme.

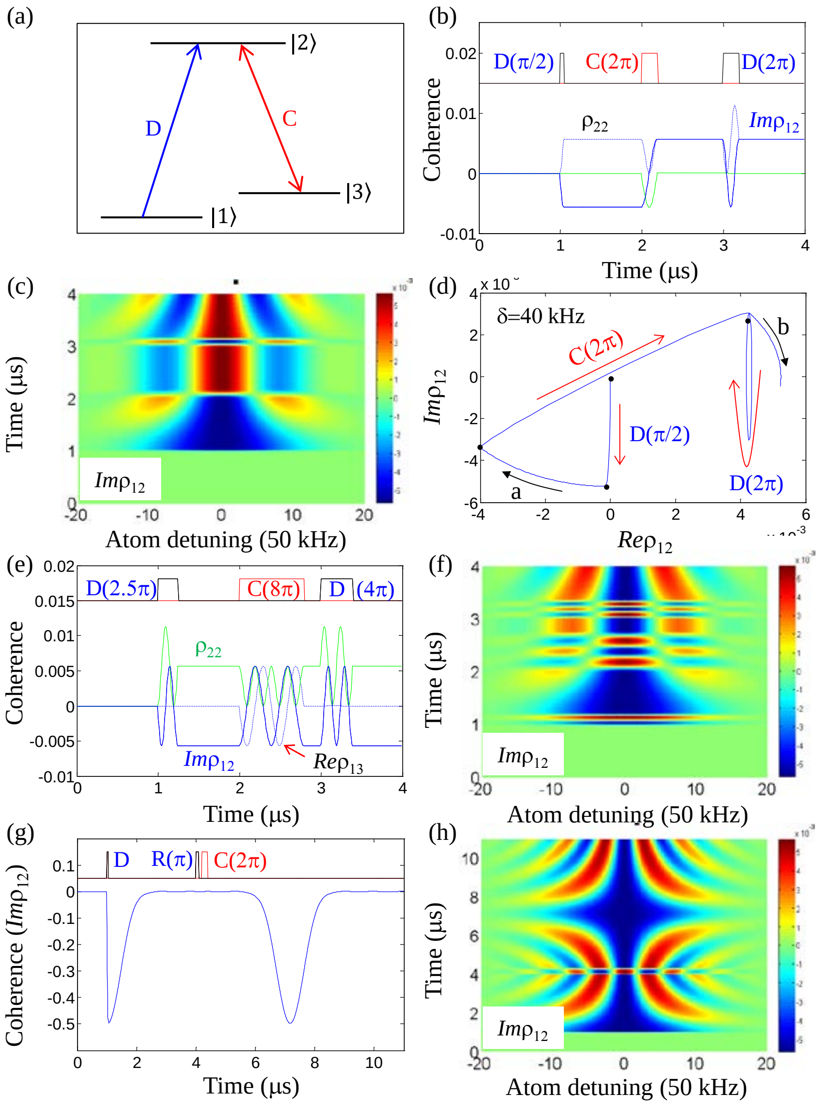

Figure S2. Rephasing vs. Controlled coherence conversion (CCC). (a) Light matter interaction scheme in a three-level system for controlled coherence conversion by C. (b) Numerical calculations of (a) for the line-centered atom (δ=0). The pulse sequence is shown in the Inset. Rabi frequency of each pulse is set by 5 MHz multiplied by 2π, where the pulse area is adjusted only by the pulse duration. The optical inhomogeneous width (Gaussian) is 1.7 MHz (FWHM), and 99.55% of the distribution is divided into 201 atom groups. All decay rates are set to zero. (c) 3D color picture for (b) for individual atoms. (d) A Bloch-vector model for a detuned atom at δ=40 kHz, showing CCC-induced coherence inversion. (e) and (f) One-photon coherence vs. two-photon coherence. (g) and (h) CCC-induced coherence inversion applied to a two-pulse photon echo.

Figure S3 shows numerical calculations of CCC by using a single 2π-control pulse C for the present controlled ac Stark echo (CASE) protocol. The inset of Fig. S3(a) shows the pulse sequence of D, AC1, R1, AC2, R2, and C. As shown in Fig. S3(a) and (b), the control 2π pulse C inverts the system coherence via Rabi flopping through a third (an auxiliary spin) state, resulting in an emissive echo in a double rephasing scheme. Although there is no population change in the excited state (see ρ_{22} in Fig. S3(b)), the coherence ρ_{12} is completely inverted as shown in Figs. S3(c) and (d): $\rho_{12} \xrightarrow{C(2\pi)} -\rho_{12}$. This coherence inversion is the key mechanism of CCC to make absorptive echoes in the double rephasing scheme emissive.

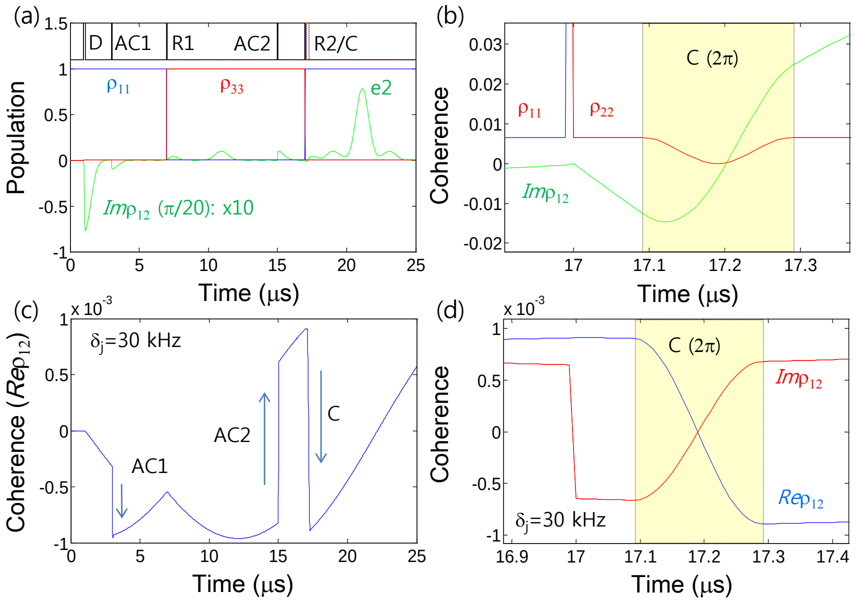

Figure S3. Population change for CASE of Fig. 5. The pulse sequence is the same as in Fig. 4. (a) CASE for a weak data pulse: $\Phi_D = \pi/20$. (b) Controlled coherence conversion by C. (c) Phase shift by AC and C. (d) Coherence inversion by C. (a)-(b) $Im\rho_{12}$ is overall coherence. (c)-(d) Individual coherence of a detuned atom at δ_j=30 kHz. Δ_{inh}=1 MHz. All other parameters are the same as in Fig. 5.

Figure S4 shows numerical calculations of a test version of Fig. 5 using a Raman system. The pulse sequence is shown in Fig. S4(a), where its interaction scheme is shown in (b). The result is shown in Fig. S4(c), where the ac Stark shift is given by $\Phi_{AC}(\tau) = \frac{\Omega_{AC}^2}{2\Delta_{AC}} \cdot \tau$ and added to each atom's detuning term δ_1 in the Hamiltonian of Eq. (13), because the density matrix calculations do not recognize the dressed state $|2-\rangle$ (or ac Stark shift). Figure S4(d) is for individual atoms' coherence evolutions of (c). Figure S4(e) is for the ac-Stark induced phase shift for the dotted line in (d). For this, the calculated ac Stark phase shift is intentionally inserted for δ_1 in the middle of calculations as mentioned above. The population change ρ_{22} in the excited state $|2\rangle$ by the ac Stark field is just ~1.4%, while the phase shift of a detuned atom is enormous as shown in Fig. S4(e). For the $\pi/2$ phase shift by AC1 for e1 erasing, the ac Stark pulse duration τ must be 3.2 μs. Figure S4(f) is for ac Stark shift calculations for several cases of Δ_{AC} with a scanning probe field for $|1\rangle - |2\rangle$ transition. As Δ_{AC} increases the dressed state ($|2-\rangle$ in the text) shift moves toward the bare state of $|2\rangle$ (see the zero-detuning region). The dotted curve is for two dressed state $|2+\rangle$ and $|2-\rangle$ at Δ_{AC}=0, which shows electromagnetically induced transparency.

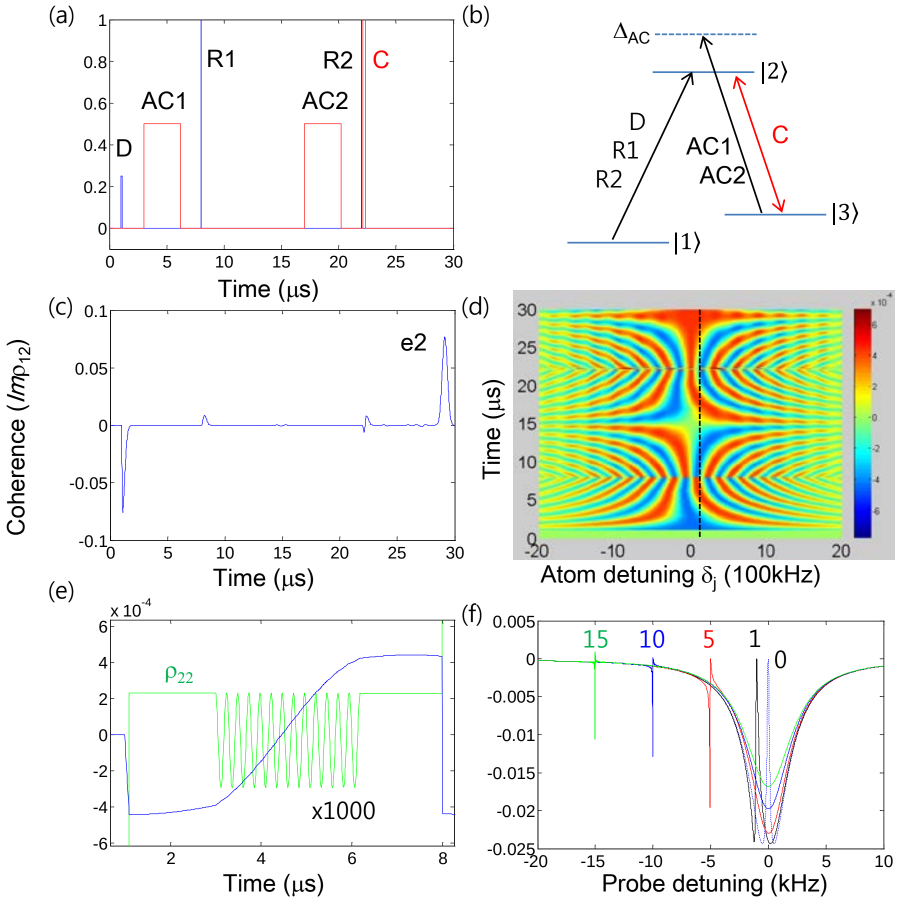

Figure S4. A test version of CASE. Δ_{inh}=1.7 MHz, Ω_{R1}=Ω_{R2}=Ω_C=10 MHz, Ω_D=250 kHz, Ω_{AC1}=Ω_{AC2}=500 kHz, Δ_{AC}=4 MHz, Φ_D=π/20. Φ_C=2π. The pulse duration of AC1 and AC2 is 3.2 μs. (a) Pulse sequence. (b) Energy level diagram. (c) Overall coherence evolution for CASE in (a). (d) Individual atom coherence evolution for (c). The dotted line is for δ_j=40 kHz in (e). (e) AC1-induced phase shift of π/2 for a detuned atom at δ_j=40 kHz. Initial magnitude of ρ_{22} before adjustment for x1000 is 3.5x10^{-5}. Thus the population change by AC1 is 1.4%, which is negligibly small. (f) The ac Stark shift for different Δ_{AC} (kHz). Dotted: Δ_{AC}=0; Black: Δ_{AC}=1; Red: Δ_{AC}=5; Blue: Δ_{AC}=10; Green: Δ_{AC}=15. Probe Rabi: Ω_P=0.1; ac Rabi: Ω_{AC}=1. Optical decay rate: γ=1. The ac Stark shift Δ_S is shifting toward the bare state |2> as Δ_{ac} increases. Homogeneous optical system is assumed for simplicity. All rates in frequency are multiplied by 2π.
